# Two years of Truvada for pre-exposure prophylaxis utilization in the US

**DOI:** 10.7448/IAS.17.4.19730

**Published:** 2014-11-02

**Authors:** Charlene Flash, Raphael Landovitz, Robertino Mera Giler, Leslie Ng, David Magnuson, Staci Bush Wooley, Keith Rawlings

**Affiliations:** 1 Section of Infectious Diseases, Department of Medicine, Baylor College of Medicine, Houston, TX, USA; 2 Division of Infectious Diseases, Department of Medicine, David Geffen School of Medicine, Los Angeles, CA, USA; 3 Drug Safety & Public Health, Gilead Sciences, Foster City, CA, USA; 4 Medical Affairs, Gilead Sciences, Foster City, CA, USA

## Abstract

**Introduction:**

Truvada^®^ (TVD) was approved in July 2012 by the US FDA for pre-exposure prophylaxis (PrEP) in combination with safer sex practices to reduce the risk of sexually acquired HIV-1 in high-risk adults. This study explores the characteristics of US PrEP users and their prescribers over the past two years.

**Materials and Methods:**

A previously described algorithm was used to identify TVD for PrEP by excluding use for HIV treatment, post-exposure prophylaxis, and off-label treatment of chronic hepatitis B. National electronic patient level data from ~55% of all US retail pharmacies that dispensed TVD between January 1, 2012 and March 31, 2014 was collected. De-identified patient-level data including prescription refill data, medical claims and patient demographics were analyzed via logistic regression to estimate the odds of change by year.

**Results:**

A total of 3253 unique individuals who started TVD for PrEP between January 1, 2012 and March 31, 2014 were included in this analysis. Women comprised 42.0% of PrEP users. Although mean age was 38.1+11.9 years, with males being significantly older (39.3+11.6) than females (36.4+12.3), 11.5% of individuals were under 25 years old. The proportion of males under 25 was 7.4% (95% CI 6.3–8.7); significantly lower than that of women, 17.2% (95% CI 15.3–19.3). New starts have increased from 293 in 2012 to 472 Q1 2014. During the 12-month period ending March 31, 2013 and March 31, 2014 the number of new starts among females dropped from 44.5% to 22.9%.

Sixty-eight percent of TVD for PrEP prescriptions were written by five specialties: internal medicine (19%), family practice (18%), infectious diseases (11%), nurse practitioners (10%) and physician assistants (10%).

**Conclusions:**

The population of TVD for PrEP users in the US nationally appears to be shifting demographically. It continues to be initiated mostly by primary care providers. Over a two-year period new starts of Truvada for PrEP have increased considerably among males. While the overall proportion of female users decreased between Q1 2012 and Q1 2014, females that started on PrEP are younger than males. More community-level data on PrEP usage will be helpful in informing local efforts to integrate PrEP in HIV prevention messaging and services.

**Table 1:** 

New TVD for PrEP starts	Overall	Q1–Q2 2012	Q3–Q4 2012	Q1–Q3 2013	Q4 2013 Q1 2014
Unique PrEP users	3253	603	713	1057	880
Mean age in years	38.1	38.4	38.7	37.8	37.7
% Younger <25 y/o	11.5	13.9	9.8	11.9	10.8
% Female	41.9	53.9	47.3	44.5	26.7
% Females < 25 y/o	17.2	17.5	14.2	18.1	19.1

